# An LDH-based prognostic model for extensive-stage small-cell lung cancer patients treated with chemo-immunotherapy and consolidative thoracic radiotherapy

**DOI:** 10.3389/fendo.2026.1836555

**Published:** 2026-05-20

**Authors:** Yunuo Zhao, Yuchi Zou, Shengxin Zhang, Yu Zhang, Xueyan Zhou, Shen Li, Yuwen Zhou

**Affiliations:** 1 Department of Biotherapy, Cancer Center and State Key Laboratory of Biotherapy, West China Hospital, Sichuan University, Chengdu, Sichuan, China; 2 Outpatient Department, West China Hospital, Sichuan University, Chengdu, Sichuan, China; 3 Department of Colorectal Cancer Center, West China Hospital, Sichuan University, Chengdu, Sichuan, China

**Keywords:** chemo-immunotherapy, consolidative thoracic radiotherapy, LDH (lactate dehydrogenase), nomogram, small-cell lung cancer

## Abstract

**Background:**

Extensive-stage small-cell lung cancer (ES-SCLC) is a highly aggressive malignancy with poor long-term survival despite multimodal therapy. Endocrine and metabolic alterations have been increasingly recognized as contributors to tumor progression and potential prognostic indicators. Lactate dehydrogenase (LDH), a readily available biomarker reflecting tumor metabolic activity, has been associated with outcomes in various cancers. However, its prognostic significance in ES-SCLC patients receiving combined chemo-immunotherapy and consolidative thoracic radiotherapy remains unclear.

**Methods:**

Between 2019 and 2025, 81 stage III-IV ES-SCLC cases treated with chemo-immunotherapy and subsequent consolidative chest radiation were retrospectively reviewed. Clinical variables and blood biomarkers were collected. To evaluate prognosis, we employed Kaplan-Meier curves and Cox proportional hazards modeling. Having identified serum LDH as an autonomous prognostic marker through multivariate analysis, we categorized it using a clinical threshold of 250 U/L for in-depth study. Subsequently, we developed a prognostic nomogram for estimating OS at 1- and 2- years. Time-dependent ROC analysis conducted at the 12- and 24-mo nth marks was utilized to evaluate the model’s discriminative power.

**Results:**

In total, 81 patients were included in the final analysis. Multivariable Cox regression demonstrated that serum LDH was independently associated with poorer overall survival (hazard ratio [HR] 1.006 per 1 U increase, 95% confidence interval [CI] 1.003–1.009; *p* < 0.001). Kaplan–Meier analysis using a clinically defined LDH threshold demonstrated significantly reduced overall survival among patients with elevated LDH levels (>250 U/L) compared with those with lower LDH values (≤250 U/L; p=0.0012). The prognostic nomogram incorporating LDH, AJCC stage, the systemic immune-inflammation index (SII), and immunotherapy status demonstrated moderate discriminative ability, with an area under the curve for OS of 0.731 at 12 months and 0.694 at 24 months.

**Conclusions:**

Our analysis indicates that serum LDH retains independent prognostic relevance for overall survival among ES-SCLC patients treated with combined chemo-immunotherapy and consolidative thoracic radiotherapy. The nomogram integrating LDH provided 1- and 2-year OS predictions, which may facilitate risk stratification and guide personalized management in the era of chemo-immunotherapy.

## 1 Introduction

Small-cell lung cancer (SCLC) represents an aggressive subtype of lung cancer with rapid tumor progression, early metastatic spread, and an overall unfavorable prognosis (1). More than two-thirds of patients present with metastatic extensive-stage disease (1, 2). For decades, platinum–etoposide chemotherapy constituted the backbone of first-line treatment for extensive-stage small-cell lung cancer (ES-SCLC), often in combination with additional locoregional or prophylactic strategies in selected patients. Despite high initial response rates, durable clinical benefits were limited (1, 2). More recently, the integration of immune checkpoint inhibitors (ICIs) into first-line platinum–etoposide–based regimens has redefined the standard of care for ES-SCLC (3). Moreover, consolidative thoracic radiotherapy (TRT) for patients with residual intrathoracic disease has yielded additional survival benefits in the chemo-immunotherapy era (4). Despite these advances, ES-SCLC outcomes remain heterogeneous. While a subset of patients achieves prolonged remission, most relapse early and the 5-year survival in extensive-stage disease remains notably low (4). This variability underscores the need for robust prognostic biomarkers to guide personalized treatment strategies.

Effective biomarkers in ES-SCLC have been elusive. Classic prognostic factors include tumor stage and performance status (5), but molecular predictors have not translated well to clinical practice. Notably, unlike non-small cell lung cancer, ES-SCLC lacks reliable genomic or immunohistochemical markers because PD-L1 expression is low and not clearly predictive, and high tumor mutational burden has shown inconsistent utility (6). In this context, simple blood-based biomarkers are attractive for risk stratification. Serum Lactate dehydrogenase (LDH) is a metabolic enzyme associated with tumor glycolytic activity (the Warburg effect) and overall tumor burden, and elevated LDH levels have consistently been linked to poor prognosis across multiple solid malignancies (7). In ES-SCLC, LDH is among the most important prognostic factors alongside stage and performance status, and high pretreatment LDH levels have been associated with significantly shorter survival (7–9). In parallel, blood count–derived inflammatory indices, including the systemic immune-inflammation index (SII), have gained recognition for their prognostic relevance in lung cancer (9–11). Recent studies indicate that a high SII correlates with worse outcomes in ES-SCLC, reflecting an immunosuppressive tumor microenvironment (12). This association is especially pronounced in patients with extensive-stage disease. However, the prognostic role of LDH in the specific setting of modern combined chemo-immunotherapy followed by TRT has not been well established. There is a paucity of data on whether these readily available biomarkers can stratify outcomes for patients receiving this intensified treatment approach.

Here, we present a retrospective cohort study evaluating prognostic factors in advanced ES-SCLC patients treated with first-line chemo-immunotherapy followed by consolidative thoracic radiotherapy. We focused on the prognostic value of baseline serum LDH, in addition to other clinical and inflammatory indices. We further constructed a multivariable prognostic model and nomogram incorporating LDH and key clinical variables to predict overall survival. The clinical variables included AJCC stage, SII, and maintenance immunotherapy status. Our aim was to develop a simple, accessible risk stratification tool for ES-SCLC in the immunotherapy era and to assess its potential utility for personalized therapy decision-making.

## 2 Methods

### 2.1 Study design and patients

**Figure 1:**
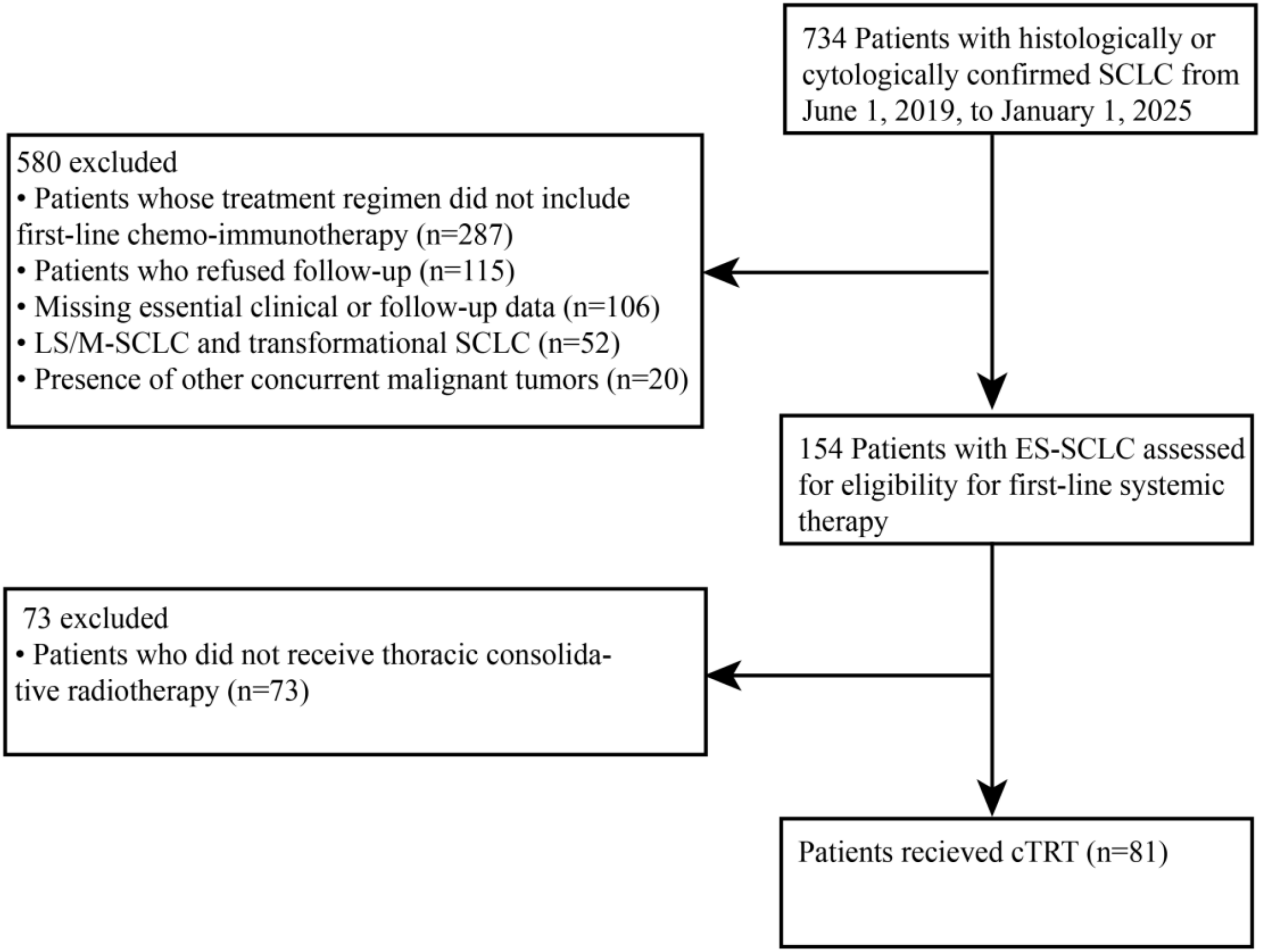
Study flow chart.

This retrospective analysis included consecutive patients diagnosed with ES-SCLC, defined as AJCC stage III-IV disease unsuitable for curative-intent radiotherapy, who received first-line chemo-immunotherapy followed by consolidative thoracic radiotherapy at West China Hospital between June 2019 and January 2025. The study flowchart is schematically illustrated in **Figure 1**. The study was performed in compliance with the principles of the Declaration of Helsinki and approved by the Institutional Ethics Committee of West China Hospital (approval No. 2025-427). The requirement for informed consent was waived owing to the retrospective nature of the study.

### 2.2 Data collection and endpoints

Eligible patients had pathologically confirmed ES-SCLC, AJCC 8th Edition stage III-IV disease, and received first-line combined chemo-immunotherapy followed by consolidative thoracic radiotherapy (TRT). Patients were included if they completed at least the induction phase of chemo-immunotherapy and subsequent TRT, with available baseline laboratory data. All patients underwent first-line treatment with platinum-etoposide chemotherapy in combination with an immune checkpoint inhibitor (atezolizumab or durvalumab). After 4-6 cycles of chemo-immunotherapy, those without disease progression underwent TRT to the primary thoracic disease (typical dose 30-60 Gy in 10-30 fractions, at physician discretion). Maintenance immunotherapy was defined as continuing ICI beyond the induction period. The decision to continue or stop ICI after chemotherapy was individualized based on response and toxicity.

Demographic characteristics and clinical variables were retrospectively extracted from the electronic medical record system. Demographic information extracted from the electronic medical record included sex, age at diagnosis, and smoking status. Clinical parameters comprised Eastern Cooperative Oncology Group performance status (ECOG PS), number and sites of metastatic lesions, AJCC stage, prior prophylactic cranial irradiation (PCI), and response to initial treatment. Treatment-related variables included receipt of maintenance immunotherapy and the number of immunotherapy maintenance cycles. Anthropometric and laboratory parameters collected at baseline included body mass index (BMI), serum LDH, systemic immune-inflammation index (SII), prognostic nutritional index (PNI), and advanced lung cancer inflammation index (ALI). Baseline laboratory parameters were derived from the latest available tests performed within 3 weeks before the start of synchronous chemo-immunotherapy. Tumor response to initial treatment was evaluated according to standard radiological assessment and categorized as complete response (CR), partial response (PR), or stable disease (SD). Treatment response was assessed by RECIST 1.1 criteria: all patients included had at least stable disease or better after induction therapy (as only those went on to TRT). Adverse events were graded per CTCAE v5.0, with a focus on immune-related and radiation-related toxicities. Baseline demographic and clinical characteristics of the study cohort are presented in **Table 1**.

**Table 1:** Baseline characteristics of patients receiving synchronous chemo-immunotherapy plus thoracic consolidative radiotherapy.

Characteristics	No. of patients (%) or median (IQR)
Gender	
Male	74 (91.4)
Female	7 (8.6)
Age (years)	65 (58-71)
ECOG PS	
0-1	77 (95.1)
≥2	4 (4.9)
Smoking history	62 (76.5)
Number of metastatic sites	
1	25 (30.9)
2	15 (18.5)
3-4	5 (6.1)
AJCC	
III	32 (39.5)
IV	49 (60.5)
Locations of metastases	
Brain	8 (9.9)
Liver	15 (18.5)
Bone	15 (18.5)
Other	38 (46.9)
Previous treatment	
PCI	22 (27.2)
Immunotherapy maintenance	52 (64.2)
Immunotherapy maintenance cycle ≤ 6	34 (42.0)
Immunotherapy maintenance cycle > 6	18 (22.2)
BMI (kg/m²)	23.11 (21.02–25.65)
Albumin (g/L)	42.55 (39.85-44.98)
Glucose (mmol/L)*	5.37 (4.96-6.63)
Globulin (g/L)*	25.65 (22.77-28.65)
A/G ratio*	1.62 (1.48-1.88)
LDL (mmol/L)*	2.57 (1.75–3.10)
HDL (mmol/L)*	1.16 (1.00-1.32)
Total cholesterol (mmol/L)*	4.42 (3.39-4.84)
LDH (U/L)*	226.5 (179.00–278.25)
SII*	602.6 (407.96–861.88)
PNI*	50.15 (47.16–53.74)
ALI*	37.95 (24.70–48.70)
Response to initial treatment	
PR/CR	71 (87.7)
SD	10 (12.3)

*The proportion of missing values for each variable was less than 30%.

BMI, body mass index; LDH, lactate dehydrogenase; PCI, prophylactic cranial irradiation; SII, systemic immune-inflammation index; PNI, prognostic nutritional index; ALI, advanced lung cancer inflammation index; PR, partial response; CR, complete response; SD, stable disease.

### 2.3 Outcomes and definitions

The primary endpoints of the study were OS, defined as the interval between initiation of treatment and death from any cause. Secondary endpoints included treatment-related adverse events (AEs), which were collected retrospectively from medical records and graded according to the Common Terminology Criteria for Adverse Events (CTCAE), version 5.0. Patients still alive were censored at the date of last contact. The survival status was updated as of August 2025 to ensure a minimum follow-up of 6 months for survivors. For analysis of LDH as a biomarker, high LDH was predefined as >250 U/L, approximately the upper limit of normal in our laboratory, and a commonly used clinical cut-off. This threshold was chosen *a priori* to facilitate clinical interpretation because values greater than 250 U/L are generally considered elevated in clinical practice. Patients were thus categorized into LDH >250 U/L vs. ≤250 U/L groups for certain analyses.

### 2.4 Inclusion and exclusion criteria

#### 2.4.1 Inclusion criteria

Patients were eligible for inclusion if all of the following conditions were satisfied:

1. aged ≥18 years;
2. pathologically confirmed extensive-stage small-cell lung cancer;
3. presence of at least one measurable, non-irradiated lesion; and
4. receipt of first-line platinum (cisplatin or carboplatin) plus etoposide chemotherapy in combination with an immune checkpoint inhibitor for a minimum of four cycles, followed by consolidative thoracic radiotherapy;

#### 2.4.2 Exclusion criteria

Patients were excluded if any of the following conditions applied:

1. absence of first-line chemo-immunotherapy combined with consolidative thoracic radiotherapy;
2. refusal to participate in follow-up;
3. incomplete essential clinical information or follow-up data;
4. diagnosis of limited-stage or mixed small-cell lung cancer, or transformed SCLC;
5. presence of other concurrent malignant tumors.

### 2.5 Statistical analysis

Patient baseline characteristics were described using appropriate descriptive statistics. The primary analysis was conducted using a complete-case approach. OS was estimated by the Kaplan-Meier method, and survival curves were compared by the log-rank test. In addition, to further explore the potential non-linear association between LDH levels and OS, restricted cubic spline (RCS) analysis was performed based on the Cox proportional hazards regression model.

Univariate analyses of potential prognostic factors were performed using Cox proportional hazards regression to calculate HR and 95% CI. Factors with a p value <0.10 in univariate analyses, together with clinically relevant variables, were subsequently incorporated into the multivariate Cox regression model. To assess the potential impact of missing data and to reduce bias, multiple imputation was performed as a sensitivity analysis. The imputation model included all variables used in the Cox regression analyses. Analyses were performed separately within each imputed dataset, and the results were pooled according to Rubin’s rules to obtain final estimates.

Nomogram was constructed to predict 1- and 2-year OS probability, assigning points to each predictor based on its Cox model coefficient. The nomogram allows calculation of a total point score for an individual patient, which corresponds to estimated survival probabilities at 12 and 24 months.

The discriminative ability of the model was evaluated using time-dependent receiver operating characteristic (ROC) analysis. The area under the ROC curve (AUC) at those time points was reported. Internal validation of the nomogram was performed using bootstrap resampling with 1,000 repetitions. Model discrimination was further quantified using the concordance index (C-index). All statistical tests were two-sided with *p* < 0.05 considered statistically significant. Analyses were carried out using R software 4.3.1.

## 3 Results

### 3.1 Baseline characteristics and clinical outcomes

#### 3.1.1 Patient characteristics

A total of 81 patients met the inclusion criteria. Baseline characteristics are summarized in **Table 1**. The cohort was predominantly male (91%) with a median age of 65 years (range 49-78). Most patients (95%) had ECOG performance status 0-1 at diagnosis. Thirty-two patients (39.5%) had stage III disease and 49 (60.5%) had stage IV disease. Among those with stage IV, common metastatic sites were liver (18.5%), bone (18.5%), and brain (9.9%), with some patients having multiple metastases. All patients received first-line platinum-etoposide chemotherapy combined with an ICI, followed by consolidative thoracic radiotherapy (cTRT). By protocol, all 81 patients underwent consolidative thoracic radiotherapy to residual thoracic disease following chemo-immunotherapy. Maintenance immunotherapy beyond induction was given in 52 patients (64.2%), whereas 29 patients (35.8%) did not continue ICI after the initial combined modality treatment, due to completion of planned cycles or treatment-related toxicity. Prophylactic cranial irradiation (PCI) was administered to 22 patients (27.2%), generally those with good response to initial therapy. At a median follow-up of 20.4 months (range 6–40 months) for surviving patients, 55 patients (67.9%) had died and 26 (32.1%) were alive at last follow-up.

#### 3.1.2 Survival outcomes

At the time of data cutoff, the median overall survival of the entire cohort was 19.2 months. The estimated 1-year and 2-year overall survival rates were 79.0% and 40.6%, respectively (**Supplementary Table 1**). Patients with stage III disease experienced significantly longer median overall survival than those with stage IV disease (27.27 vs. 16.85 months; log-rank p=0.037; **Supplementary Figure 1**).

#### 3.1.3 Adverse events

During combined chemo-immunotherapy and consolidative thoracic radiotherapy, treatment-related adverse events were common but generally manageable (**Supplementary Table 2**). Chemotherapy-related adverse events were the most frequently observed. Myelosuppression occurred in 29 patients (35.8%), of whom 21 (25.9%) experienced grade I–II events and 8 (9.9%) developed grade III–IV toxicity. Abnormal liver function was observed in 14 patients (17.3%), with grade III–IV events occurring in only 2 patients (2.5%). Abnormal coagulation was reported in 9 patients (11.1%), including 4 cases (4.9%) of grade III–IV toxicity. Pulmonary infection occurred in 17 patients (21.0%), with 4 patients (4.9%) experiencing grade III–IV events. Radiotherapy-related adverse events were less frequent. Radiation pneumonitis was observed in 15 patients (18.5%), including 5 cases (6.2%) of grade III–IV severity. Radiation esophagitis occurred in 10 patients (12.3%), with only 1 patient (1.2%) developing grade III–IV toxicity. Immune-related adverse events were generally mild to moderate. Immune pneumonitis was reported in 9 patients (11.1%), with 2 cases (2.5%) of grade III–IV events. Immune myocarditis was observed in 4 patients (4.9%), all of which were grade I–II. Immune thyroiditis occurred in 10 patients (12.3%), including 2 patients (2.5%) with grade III–IV toxicity. Immune-related rash was reported in 8 patients (9.9%), with only 1 case (1.2%) of grade III–IV severity. No treatment-related deaths were observed.

### 3.2 Cox regression analysis for overall survival

**Figure 2:**
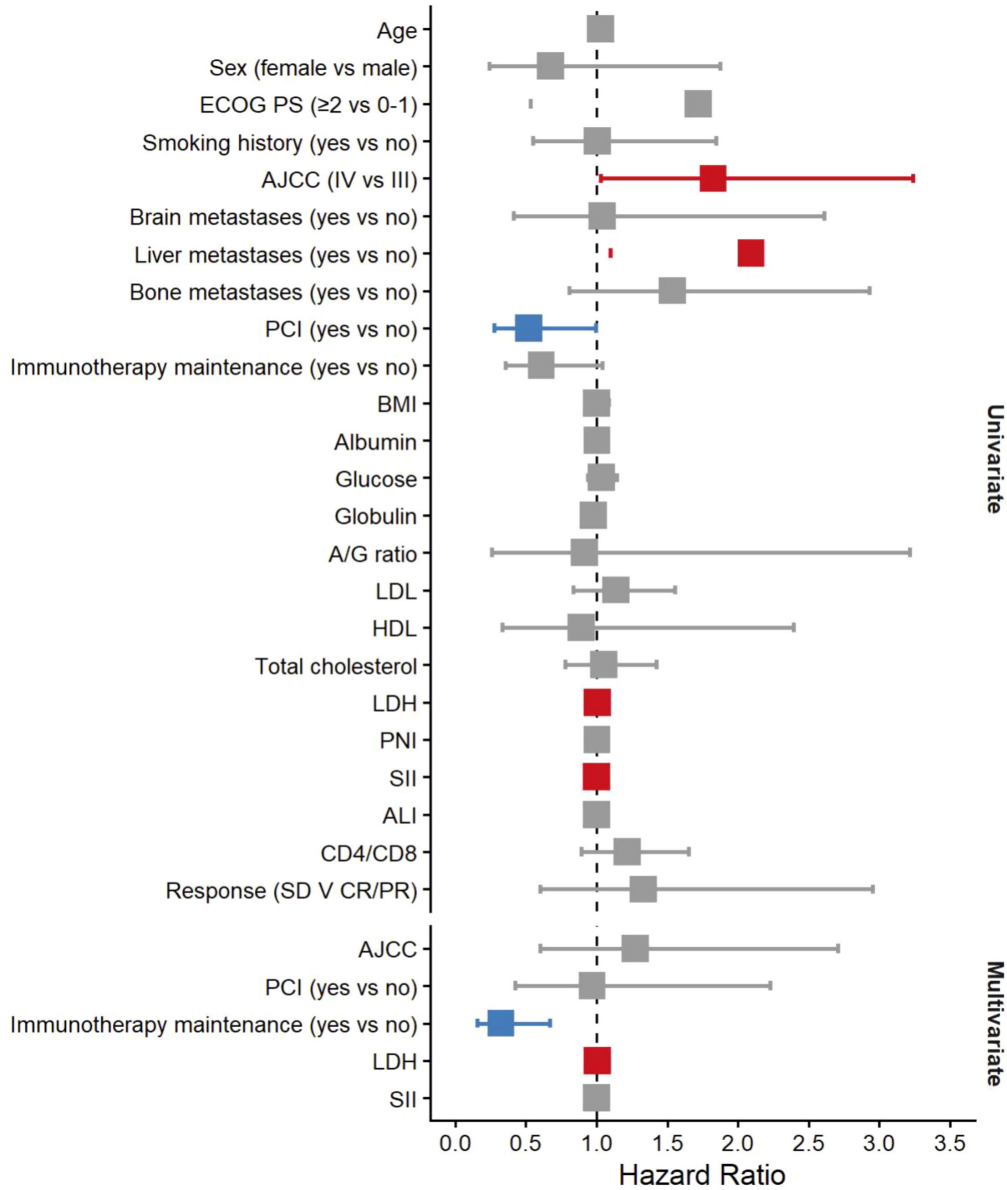
Forest plot of univariate and multivariate Cox regression analyses for overall survival.

We next performed univariate and multivariate Cox proportional hazards analyses to identify independent predictors of OS (**Table 2**). **Figure 2** presents the forest plot of hazard ratios for candidate variables in univariate and multivariate models. In univariate analysis, factors significantly associated with shorter OS included: advanced AJCC stage (stage IV vs III, HR 1.825, 95% CI 1.028–3.241, *p* = 0.039), presence of liver metastases (HR 2.094, 95% CI 1.098-3.993, *p* = 0.025), PCI (HR 0.522, 95% CI 0.275-0.992, *p* = 0.047), and higher LDH (analyzed as a continuous variable, per 1 U/L increase, HR 1.004, 95% CI 1.001-1.007, p<0.001). Among metabolism- and endocrine-related indicators, only LDH showed a significant association with OS, whereas the remaining markers were not significantly associated with survival. Elevated SII was also associated with worse survival on univariate analysis (HR 1.001 per 1 unit increase, p=0.04). Other variables such as age, sex, ECOG performance status, smoking history, bone or brain metastases, and body mass index were not significantly correlated with OS in this cohort (*p*>0.1 for each). Notably, maintenance immunotherapy showed a strong trend: patients who did not continue immunotherapy had a higher risk of death (HR 1.64, 95% CI 0.96-2.81) with *p* = 0.07 in univariate analysis.

**Table 2:** Univariate and multivariate Cox regression analyses for overall survival.

Variables	Univariate			Multivariate		
	HR	95% CI	*P*	HR	95% CI	*P*
Age	1.030	0.993-1.068	0.113			
Sex (female vs male)	0.675	0.244-1.871	0.450			
ECOG PS (≥2 vs 0-1)	1.719	0.535-5.526	0.363			
Smoking history (yes vs no)	1.006	0.548-1.848	0.984			
AJCC (stage IV vs III)	1.825	1.028-3.241	**0.039**	1.274	0.599-2.708	0.529
Brain metastases (yes vs no)	1.038	0.413-2.610	0.937			
Liver metastases (yes vs no)	2.094	1.098-3.993	**0.025**			
Bone metastases (yes vs no)	1.536	0.805-2.932	0.193			
PCI (yes vs no)	0.522	0.275-0.992	**0.047**	0.969	0.422-2.229	0.942
Immunotherapy maintenance (yes vs no)	0.609	0.356-1.042	0.070	0.324	0.156-0.672	**0.002**
BMI	1.000	0.921-1.085	0.992			
Albumin	1.003	0.998-1.008	0.297			
Glucose	1.034	0.936-1.143	0.507			
Globulin	0.978	0.915-1.046	0.523			
A/G ratio	0.913	0.259-3.214	0.888			
LDL	1.138	0.835-1.552	0.412			
HDL	0.894	0.334-2.395	0.824			
Total cholesterol	1.053	0.779-1.423	0.737			
LDH	1.004	1.001-1.007	**<0.001**	1.006	1.003-1.009	**<0.001**
PNI	1.003	0.997-1.008	0.316			
SII	1.001	1.000-1.001	**0.042**	1.0004	1.000-1.001	0.199
ALI	0.998	0.981-1.015	0.805			
CD4/CD8	1.216	0.894-1.654	0.212			
Response (SD vs CR/PR)	1.333	0.602-2.952	0.479			

HR, hazard ratio; CI, confidence interval. The bold values indicate statistical significance, specifically variables with P < 0.05.

In the multivariate Cox model, LDH and maintenance immunotherapy status emerged as independent prognostic factors after adjusting for other covariates. Baseline LDH, analyzed as a continuous variable, retained a highly significant effect (adjusted HR 1.006 per U/L, 95% CI 1.003–1.009, *p* < 0.001). This corresponds to roughly a 60% increase in mortality risk for every 100 U/L increase in LDH. Maintenance immunotherapy was an independent protective factor: receiving ICI maintenance was associated with a significantly lower risk of death (HR 0.324, 95% CI 0.156–0.672, p = 0.002) compared to those who discontinued treatment, indicating that ongoing immunotherapy was associated with a 68% reduction in the hazard of death. Liver metastasis was not retained in the multivariate model once stage was included.

To assess the robustness of these findings in the presence of missing data, a sensitivity analysis was performed using multiple imputation. In the imputed dataset, LDH remained associated with overall survival (HR 1.004, 95% CI 1.0003–1.0079, p=0.037), supporting the stability of its prognostic effect across different analytical approaches. In contrast, the association between maintenance immunotherapy and overall survival was attenuated and did not reach statistical significance in the imputed analysis (HR 0.543, 95% CI 0.273–1.079, p=0.079). Other covariates demonstrated comparable or reduced effect estimates after imputation. Overall, these findings suggest that the prognostic impact of LDH is robust to different missing data assumptions, whereas some clinical covariates may be more sensitive to sample variability (**Supplementary Table 4**).

### 3.3 Prognostic value of baseline LDH

**Figure 3:**
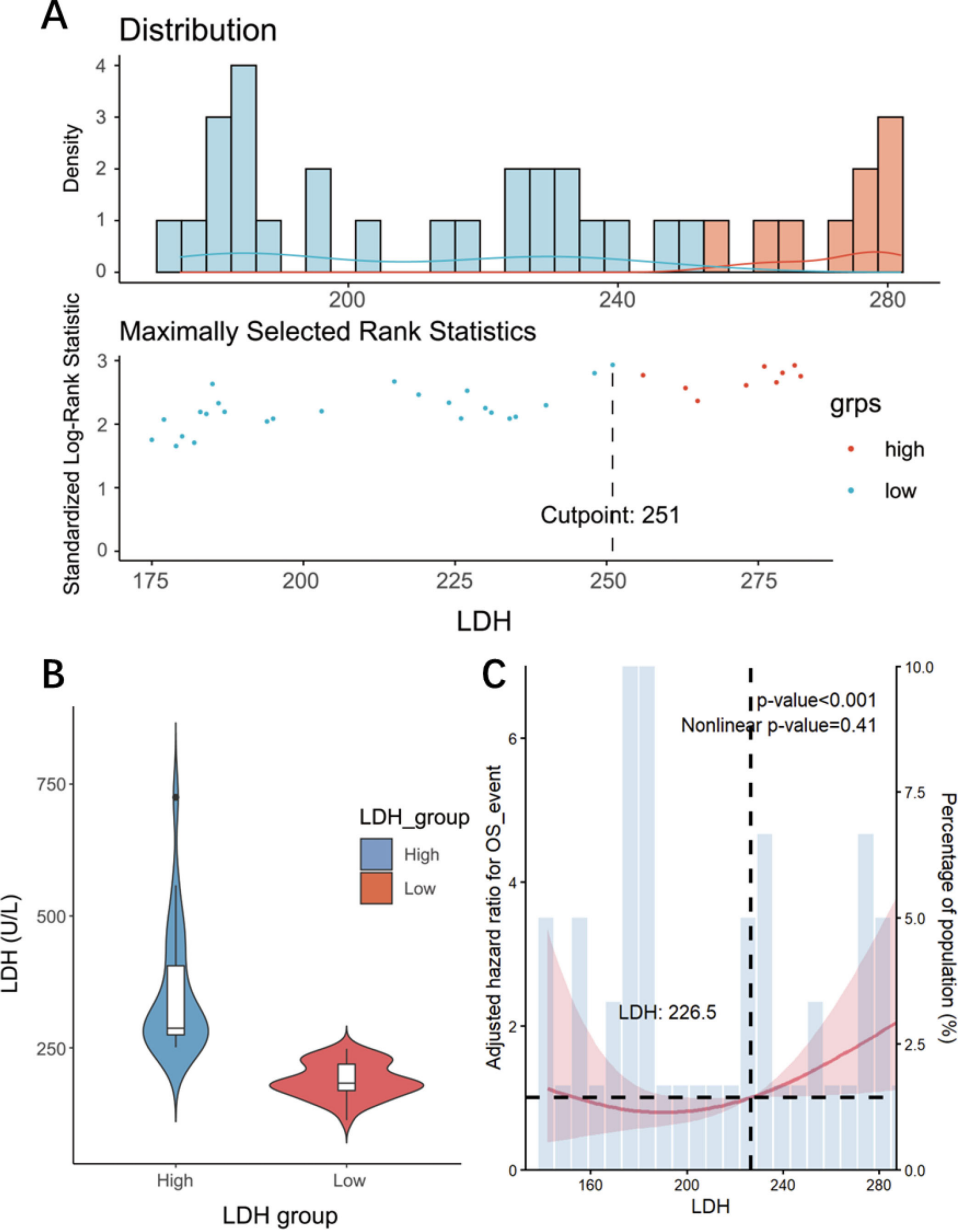
Baseline serum LDH distribution and group stratification **(A)** density distribution of baseline LDH values and standardized log-rank statistics across candidate cut-points. **(B)** violin plot showing baseline LDH distributions in the low (≤250 U/L) and high (>250 U/L) LDH groups (p < 0.001). **(C)** RCS analysis of the association between baseline LDH levels and overall survival OS.

Baseline LDH values were available in 60 patients, whereas 21 patients had missing LDH data. We defined 250 U/L as the cut-off value for LDH because it represents the upper limit of the standard clinical reference range (typically 120–250 U/L) in our institution. The median LDH level was 226.5 U/L (range 113-725), and 23 of 60 patients (38.3%) had LDH >250 U/L at baseline. **Figure 3A** illustrates the distribution of baseline LDH values and an exploratory cut-point scanning analysis. The upper panel shows the density distribution of LDH levels across the cohort. The lower panel displays the standardized log-rank statistics across a range of candidate LDH thresholds derived from the surv_cutpoint algorithm, indicating how survival separation varied with different cut-points (13, 14). Although the maximal rank statistic was observed at an LDH value of 251 U/L, this analysis was performed for descriptive purposes only. The LDH cut-off applied in all subsequent analyses was prespecified at 250 U/L based on routine clinical reference standards, and the proximity of the exploratory peak to this value suggests consistency between the clinical threshold and the underlying survival patterns in this cohort. **Figure 3B** illustrates the distribution of serum LDH levels across the predefined LDH strata. Patients with LDH values of 250 U/L or lower were categorized into the low-LDH group, whereas those with LDH levels exceeding 250 U/L comprised the high-LDH group. A significant difference in LDH concentrations was observed between the two groups (*p* < 0.001). RCS analysis suggested that the overall association between LDH and OS was statistically significant (overall p<0.001), indicating that LDH levels were closely associated with survival. However, the test for non-linearity was not statistically significant (nonlinear p=0.41). Therefore, although the RCS curve visually showed a U-shaped pattern, there was insufficient statistical evidence to support a significant non-linear association between LDH levels and the risk of death (**Figure 3C**).

**Figure 4:**
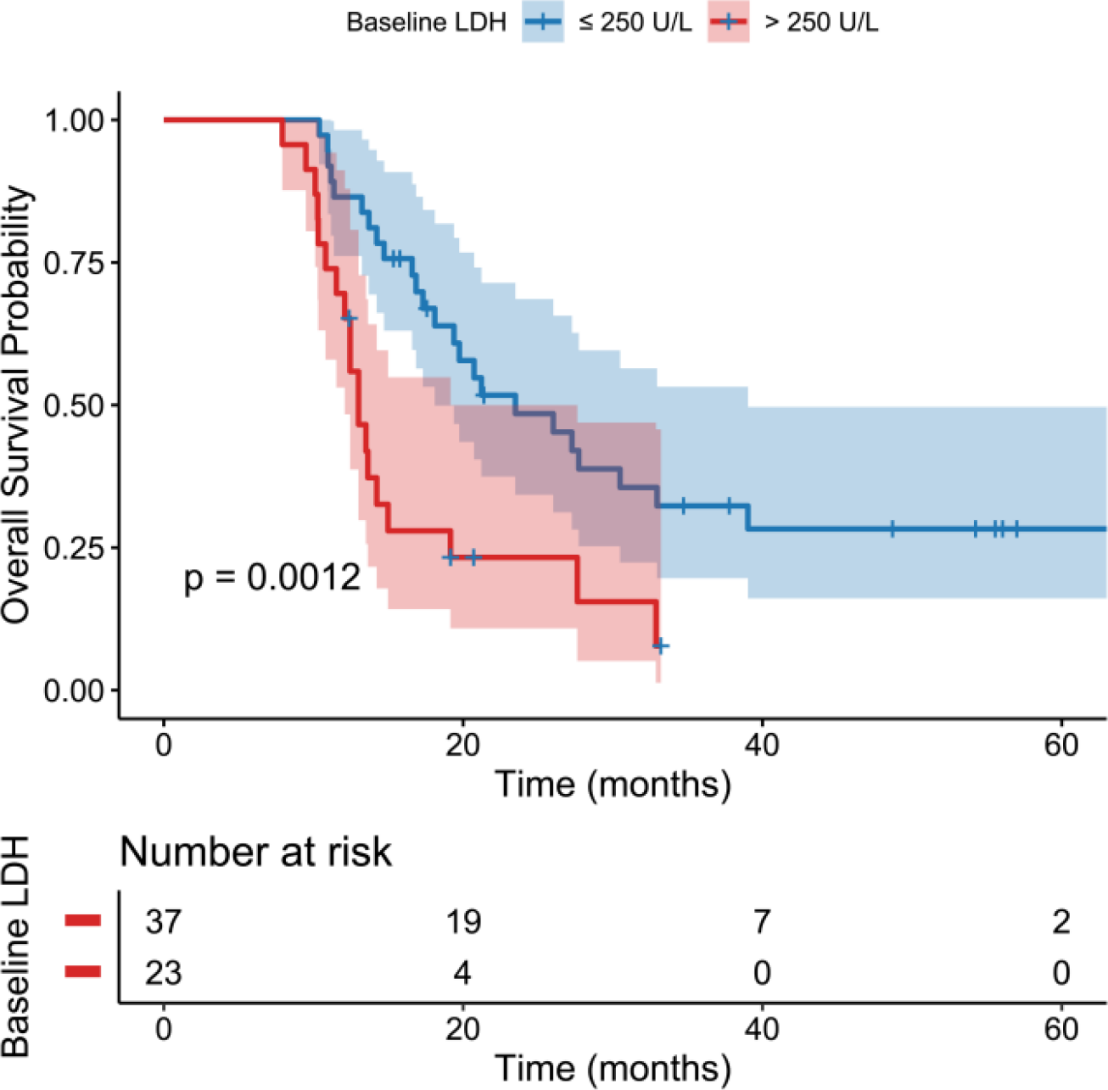
Kaplan-Meier curves of overall survival stratified by baseline LDH levels (≤250 U/L vs >250 U/L).

Importantly, LDH emerged as a strong prognostic discriminator. On Kaplan-Meier analysis stratified by LDH, patients with elevated LDH >250 U/L had markedly worse survival than those with LDH ≤250 U/L. The median OS in the high LDH group was 12.09 months, compared to 18.10 months in the low LDH group (**Supplementary Table 3**). The survival curves separated early, and the difference was statistically significant (log-rank *p* < 0.001) (**Figure 4**). Thus, a baseline LDH above 250 U/L portended a significantly shorter OS, confirming the adverse prognostic value of LDH in this treatment setting.

### 3.4 Nomogram for overall survival prediction

**Figure 5:**
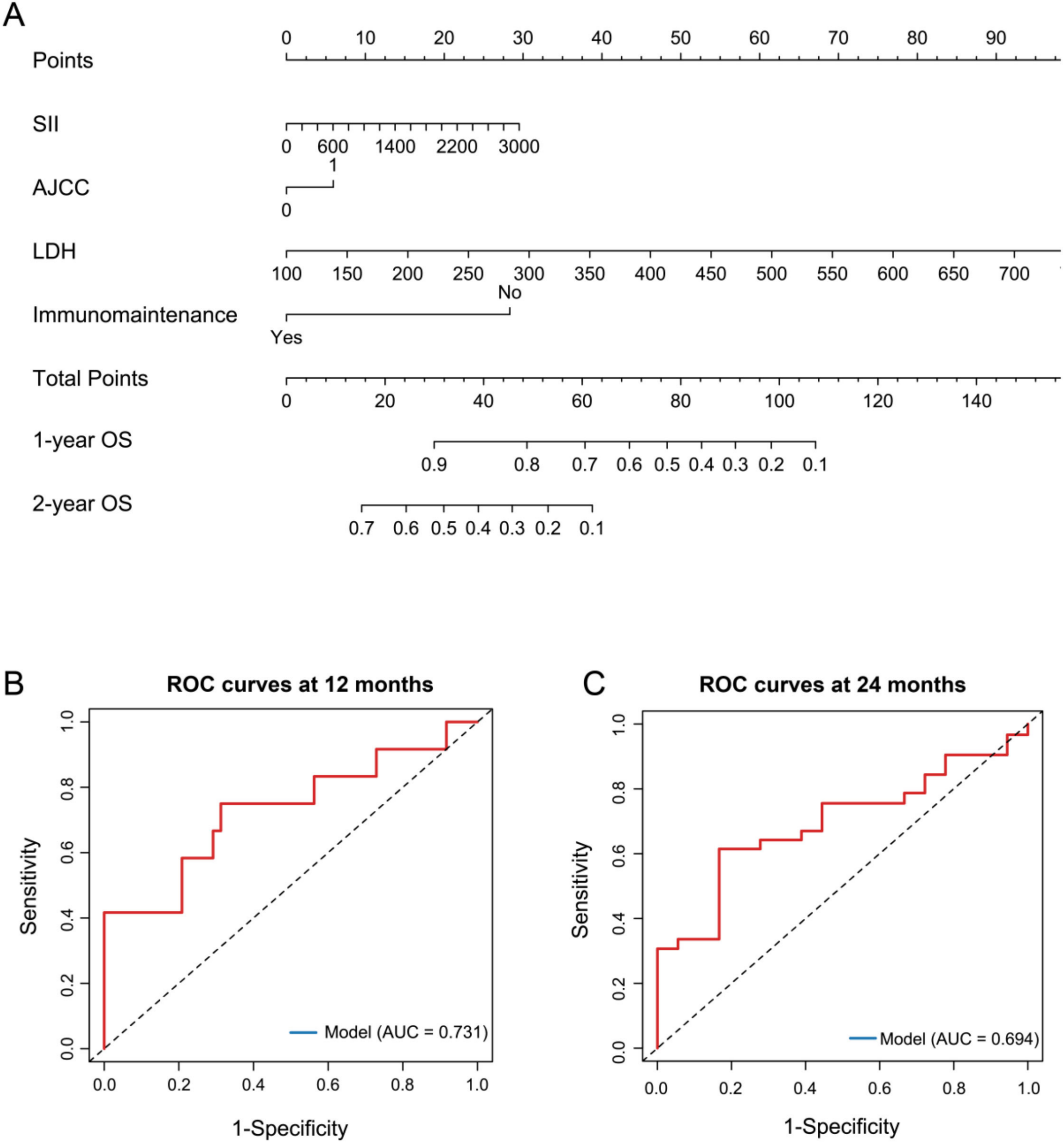
Nomogram for overall survival prediction and its discriminatory performance **(A)** prognostic nomogram to estimate 1- and 2-year overall survival probabilities; **(B, C)** time-dependent ROC curves of the nomogram for overall survival prediction at 12 months (AUC = 0.731) and 24 months (AUC = 0.694).

Guided by the multivariate Cox regression findings, serum LDH and maintenance immunotherapy were retained as independent predictors of overall survival and were incorporated as key components of the prognostic nomogram. In addition, AJCC stage was incorporated into the model owing to its well-established clinical relevance in prognostic stratification of ES-SCLC, despite not retaining independent statistical significance after adjustment (15). SII was also included in the nomogram, as it demonstrated a trend toward prognostic relevance in univariate analysis and represents an integrative marker of systemic inflammatory status that may complement metabolic and staging information (16). A prognostic nomogram incorporating LDH, maintenance immunotherapy status, AJCC stage, and SII was developed to predict overall survival at 1- and 2- years (**Figure 5A**). The nomogram assigns individualized point values to each variable, allowing calculation of total risk scores and corresponding survival probabilities. Model discrimination was assessed using time-dependent receiver operating characteristic analysis. The area under the ROC curve for overall survival prediction was 0.731 at 12 months and 0.694 at 24 months (**Figures 5B, C**), indicating good short- to intermediate-term prognostic accuracy of the model. Internal validation was performed using bootstrap resampling with 1,000 repetitions. The apparent concordance index (C-index) of the nomogram was 0.708, while the optimism-corrected C-index was 0.687, suggesting moderate discriminative ability and acceptable model stability.

## 4 Discussion

In the present analysis, serum LDH emerged as a clinically relevant indicator of prognosis in ES-SCLC patients treated with chemo-immunotherapy followed by consolidative thoracic radiotherapy. Higher baseline LDH was associated with significantly shorter overall survival, even after adjusting for disease stage and other factors. Patients with LDH above 250 U/L had a markedly poor outcome despite aggressive combined-modality treatment. These findings highlight that LDH, a simple and inexpensive laboratory test, may help stratify risk among patients with advanced ES-SCLC and complement traditional prognostic indicators. Our results extend evidence from earlier treatment eras and suggest that LDH remains a clinically relevant biomarker even in the context of immunotherapy.

The prognostic importance of LDH in ES-SCLC is biologically plausible and consistent with prior research. LDH is a key enzyme in anaerobic glycolysis and is often elevated in patients with high tumor burden or more aggressive disease, reflecting the Warburg effect and tumor hypoxia (17). Historically, LDH elevation has been regarded as a marker of aggressive disease biology and poor prognosis in multiple solid tumor types (18). In ES-SCLC specifically, elevated LDH was identified as a predictor of poor survival in the pre-immunotherapy era (19). He et al. reported that ES-SCLC patients with pretreatment LDH ≥215 U/L had significantly worse survival than those below that threshold, and LDH remained an independent prognostic factor along with age, performance status, and disease extent (19). Our findings align well with these data, reinforcing that LDH captures aspects of tumor aggressiveness not fully accounted for by conventional staging. Moreover, emerging evidence in the immunotherapy setting corroborates our results, as a recent meta-analysis of ES-SCLC patients treated with ICIs found that high baseline LDH correlated with poorer OS (8). This suggests that LDH may reflect a subgroup of patients with poorer outcomes despite immunotherapy-based treatment, although whether LDH is predictive of immunotherapy benefit remains unclear.

We also found that continuation of maintenance immunotherapy was associated with longer survival, although this finding may partly reflect patient selection and treatment tolerance. While this observation does not establish causality, it highlights maintenance ICI therapy as a clinically relevant factor in prognostic stratification within this treatment setting. These findings lend real-world support to the inclusion of maintenance immunotherapy in contemporary ES-SCLC management and warrant further investigation in prospective studies. It also raises a practical point that baseline LDH might help identify patients who are less likely to benefit from prolonged immunotherapy. In our high LDH subgroup, outcomes remained poor despite treatment. These patients could potentially be candidates for alternate strategies or clinical trials because their disease may be intrinsically resistant. Future studies could explore LDH as one component of an immune prognostic index to better tailor the use of immunotherapy.

The inclusion of systemic immune-inflammation index (SII) in our nomogram adds a novel dimension to the prognostic assessment. Although SII did not reach independent significance in the multivariate model (*p* = 0.19), we elected to retain it because of its known prognostic value and to improve the model’s predictive accuracy. SII is a composite marker that reflects the balance between host inflammation and immune response. Specifically, high neutrophil and platelet counts can promote tumor progression and suppress anti-tumor immunity, while lymphopenia indicates a weakened immune defense against cancer, all of which contribute to an elevated SII (20). Our cohort showed a trend toward worse survival with higher SII, consistent with prior studies (12). By integrating SII, our model acknowledges the role of the tumor–immune interaction in determining outcomes. We anticipate that as more data accumulate, inflammatory indices like SII or NLR will be increasingly incorporated into prognostic models, especially in the era of immunotherapy where host immune status is critical.

Our final model also retained AJCC stage (III vs IV), despite the fact that the present study exclusively focused on patients with extensive-stage ES-SCLC. Within this population, AJCC staging still captures meaningful heterogeneity in anatomic disease burden, particularly with respect to the presence and extent of distant metastases. Although AJCC stage did not remain statistically significant after adjustment for LDH and SII, it remains clinically intuitive that patients with stage IV disease generally have a less favorable baseline prognosis than those with stage III disease, even within the extensive-stage setting. Consistent with this notion, univariate analysis demonstrated that stage IV disease was associated with a substantially higher hazard of death compared with stage III (21). By retaining AJCC stage in the nomogram, we anchored the model in a widely accepted and clinically interpretable indicator of tumor burden.

When comparing our prognostic model to others in the literature, we find comparable performance. Our nomogram achieved 1-year AUC of approximately 0.73, indicating moderate discriminative ability in predicting survival. Wang et al. recently developed a nomogram for ES-SCLC patients receiving ICIs based on albumin, glucose-to-lymphocyte ratio (GLR), and other clinical factors; their model attained a C-index of 0.752 with AUC of approximately 0.80 at 1 year (14). The slightly higher AUC in that study may be due to the inclusion of additional tumor markers (e.g. NSE, CEA) or the larger sample size. Nonetheless, the performance of our LDH-based model is in a similar range, suggesting that a few readily available parameters can achieve reasonably accurate risk stratification. Notably, our model uses very routine labs and basic clinical information, making it highly practical. In a related study, Yuan et al. reported a prognostic nomogram for extensive-stage ES-SCLC patients receiving thoracic radiotherapy that incorporated inflammatory biomarkers and clinical factors. The conceptual overlap between the two models lends support to the robustness of biomarker-based prognostic stratification in this setting (22). This concordance builds confidence that our approach is robust. Overall, our results and others’ collectively support that LDH stands out as an independent driver of prognosis in ES-SCLC, and incorporating it with other clinical variables yields a simple yet effective stratification tool.

From a clinical perspective, the establishment of an LDH-based prognostic model has practical implications (23). First, it enables personalized risk assessment at diagnosis using data available in any clinic. Patients with extensive-stage ES-SCLC characterized by elevated LDH and higher SII tended to exhibit a less favorable survival profile, whereas those with lower LDH levels and more favorable inflammatory markers experienced comparatively improved outcomes. Second, such risk stratification could be used to tailor treatment intensity. One could hypothesize that high-risk patients with high LDH might merit additional interventions, such as enrollment in trials of novel agents upfront or the intensification of local therapy in cases of oligometastatic disease. Conversely, low-risk patients might be spared from overly aggressive approaches that increase the risk of toxicity. Additionally, knowing a patient’s risk category may influence follow-up frequency and supportive care; high-LDH patients might require closer surveillance for early relapse and preemptive palliative measures.

We acknowledge several limitations to our study. First, the sample size of 81 patients was relatively modest, and the data were retrospectively collected from a single center. This may limit the generalizability of the findings and introduces the potential for selection bias. In particular, our cohort consisted of patients who were sufficiently fit to receive both immunotherapy and thoracic radiotherapy, thereby excluding patients with very poor performance status or highly aggressive disease who may have had even worse outcomes. Second, because of the retrospective design, unmeasured confounders may have influenced survival outcomes. These may include differences in salvage treatments, variations in immune checkpoint inhibitor regimens, and other clinical factors not fully captured in the dataset. In addition, LDH data were only available for 60 patients, and some patients also had missing SII values. If patients without these laboratory measurements differed systematically from those with complete data, this may have introduced bias. For example, laboratory testing may have been omitted in patients with rapid clinical deterioration. Third, although the nomogram demonstrated moderate discriminative ability, its predictive performance should be interpreted cautiously. The AUC values of 0.731 at 12 months and 0.694 at 24 months, together with a corrected C-index of 0.687, suggest only modest predictive accuracy rather than strong prognostic performance. Therefore, the model should currently be viewed as an exploratory tool that may assist risk stratification rather than as a definitive predictive instrument for clinical decision-making. Finally, our nomogram has not yet undergone external validation. Validation in larger, independent cohorts is needed to confirm the robustness, calibration, and transportability of the model. Prospective studies would be particularly valuable to determine whether LDH and SII can provide meaningful prognostic information in real-time clinical practice. Despite these limitations, our findings suggest that even in the era of chemo-immunotherapy and consolidative thoracic radiotherapy for ES-SCLC, a simple and readily available serum biomarker such as LDH may still offer clinically relevant prognostic information.

## 5 Conclusion

In summary, serum LDH is an easily measurable biomarker that provides valuable prognostic information for small-cell lung cancer patients undergoing chemo-immunotherapy and thoracic radiotherapy. High LDH identifies a subset of patients with particularly poor outcomes despite aggressive treatment. We have presented a nomogram that combines LDH with stage, SII, and treatment variables to predict survival, enabling clinicians to stratify patients by risk and personalize treatment decisions. The model performed competently in our cohort, highlighting the potential of routine blood-based markers in guiding ES-SCLC management. Given its simplicity and cost-effectiveness, this LDH-based prognostic tool may have potential clinical utility if validated in larger prospective cohorts. Future studies should validate this model prospectively and investigate whether tailored interventions for high-risk patients with high LDH can improve their outcomes. Our work emphasizes that even in the era of modern immunotherapy, traditional biomarkers like LDH remain highly relevant and can enrich our approach to individualized care in ES-SCLC.

## Data Availability

The raw data supporting the conclusions of this article will be made available by the authors, without undue reservation.
