# Supplementary material for: An LDH-based prognostic model for extensive-stage small-cell lung cancer patients treated with chemo-immunotherapy and consolidative thoracic radiotherapy
Source: Front Endocrinol (Lausanne). 2026 May 20;17:1836555. doi: 10.3389/fendo.2026.1836555 (PMC13229745; doi:10.3389/fendo.2026.1836555)

**Supplementary Material**

**Supplementary Table 1.** Overall survival outcomes in the entire cohort

| **Outcome** | **Value** |
| --- | --- |
| **Median overall survival (months)** | **19.2** |
| 1-year overall survival rate | 79.0 % |
| 2-year overall survival rate | 40.6 % |

**Supplementary Table 2.** Adverse events during concurrent chemo-immunotherapy and consolidative thoracic radiotherapy.

| **Characteristic** | **Toal (%)** | **Grade I/II (%)** | **Grade III/IV (%)** |
| --- | --- | --- | --- |
| **Chemotherapy-related adverse events** |  |  |  |
| Myelosuppression | 29 (35.8) | 21 (25.9) | 8 (9.9) |
| Abnormal liver function | 14 (17.3) | 12 (14.8) | 2 (2.5) |
| Abnormal coagulation | 9 (11.1) | 5 (6.17) | 4 (4.9) |
| Pulmonary infection | 17 (21.0) | 13 (16.1) | 4 (4.9) |
| **Radiotherapy-related adverse events** |  |  |  |
| Radiation pneumonitis | 15 (18.5) | 10 (12.3) | 5 (6.2) |
| Radiation esophagitis | 10 (12.3) | 9 (11.1) | 1 (1.2) |
| **Immune-related adverse events** |  |  |  |
| Immune pneumonitis | 9 (11.1) | 7 (8.6) | 2 (2.5) |
| Immune myocarditis | 4 (4.9) | 4 (4.9) | 0 |
| Immune thyroiditis | 10 (12.3) | 8 (9.9) | 2 (2.5) |
| Immune rash | 8 (9.9) | 7 (8.6) | 1 (1.2) |

**Supplementary Table 3.** Overall survival according to baseline LDH stratification.

| **LDH group** | **Overall survival (months)** |
| --- | --- |
| **LDH≤250 U/L** | 18.10 |
| **LDH＞250 U/L** | 12.09 |

**Supplementary Table 4. Multivariate Cox regression analysis after multiple imputation**

| **Variable** | **HR** | **95% CI** | **P value** |
| --- | --- | --- | --- |
| AJCC | 1.861 | 0.939 – 3.686 | 0.074 |
| Immunotherapy maintenance | 0.543 | 0.273 – 1.079 | 0.079 |
| LDH | 1.004 | 1.0003 – 1.0079 | 0.037 |
| PCI | 0.605 | 0.269 – 1.359 | 0.215 |
| SII | 1.0005 | 0.9996 – 1.0013 | 0.25 |

**Supplementary Figure 1.** **Kaplan-Meier analysis of overall survival according to AJCC stage subgroup**


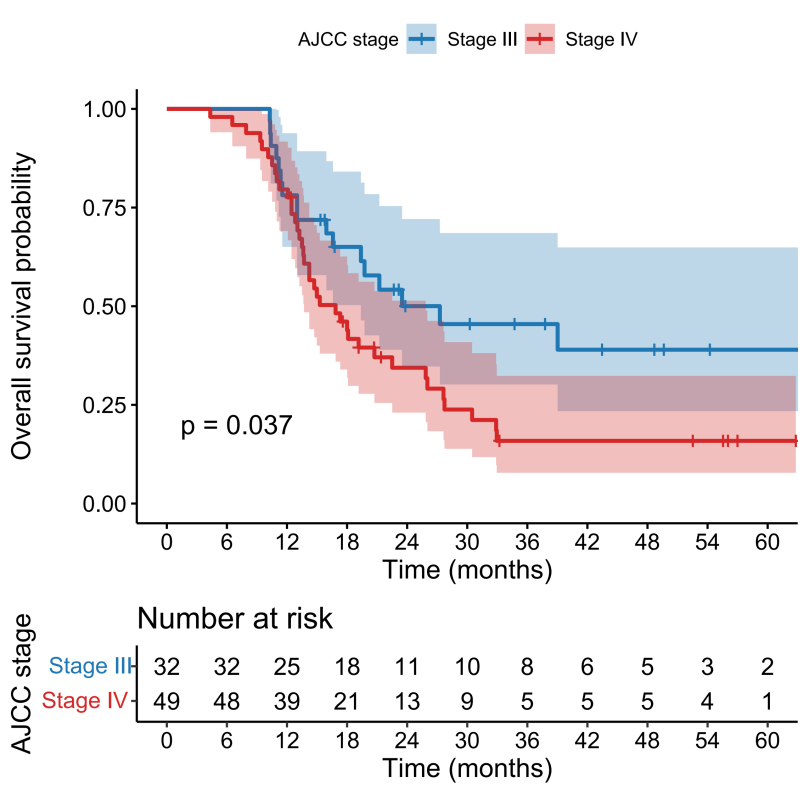

Supplement: Supplementary file 1 [file DataSheet1.docx]
